# Damage to newly synthesized proteins is a major cause of Cd(II) toxicity counteracted by proteasomes and integrated stress response in human cells

**DOI:** 10.1016/j.cbi.2026.112045

**Published:** 2026-03-18

**Authors:** Giorgiana Madalina Ursu, Anatoly Zhitkovich

**Affiliations:** Brown University, Department of Pathology and Laboratory Medicine, Providence, RI, 02903, USA

**Keywords:** Cadmium, Proteotoxicity, Proteasomes, Unfolded proteins, Protein damage

## Abstract

Cadmium (Cd) is a biopersistent metal causing cancer and toxicity in several human tissues. Cd(II) lacks DNA binding or direct redox activity and its toxicity may result from protein damage. However, it is unclear what proteins are preferentially damaged by Cd(II) and whether global or protein-specific damage underlies its main pathologies. We examined the origin and toxicological significance of the global proteotoxic stress induced by Cd (II) in human lung and kidney cells, including primary renal proximal tubule cells. In all cells, low doses of Cd(II) induced proteolytic K48-polyubiquitination and insolubility (denaturation) of proteins. Ubiquitination-inactive cells showed hyperaccumulation of Cd-denatured proteins and transient suppression of ubiquitination or proteasome activity severely impaired cell viability at otherwise nontoxic doses of Cd. Inhibition of the ubiquitin-proteasome system after Cd(II) treatments was also detrimental to cell viability, indicating ongoing protein damage. Newly synthesized polypeptides were the main source of Cd(II)-denatured proteins and inhibition of translation prevented the formation of cytosolic aggresomes with amyloid-like structures. Short-lived transcription (p53, c-MYC) or antiapoptotic (MCL1) factors were especially sensitive to unfolding/denaturation by Cd(II). Activation of integrated stress response by Cd(II) increased cell survival and lowered the burden of structurally damaged proteins although to a lesser extent than proteasome activity. Our findings identified newly synthesized proteins as the major target of toxic damage by Cd(II) in kidney proximal tubule and other cells and revealed a high vulnerability of short-lived proteins. Ubiquitin-proteasome system was critically important for removal of damaged proteins and Cd(II) tolerance by human cells.

## 1. Introduction

Cadmium (Cd) is a toxic environmental pollutant that poses significant global health risks to humans and animals [1,2]. Environmental release of Cd occurs during natural biomass combustion and volcanic eruptions as well as from numerous anthropogenic sources. Global soil pollution with overly high levels of toxic metals is estimated to impact more than 1 billion people, with Cd being the most widespread metal contaminant [3]. The major route of Cd exposure in the general population is consumption of contaminated food with a secondary contribution from cigarette smoking. Cd is a biopersistent metal that is eliminated from the human body extremely slowly (25–30 years half-life) [1,2]. Inhalation exposures to Cd lead to pulmonary damage and increase the risk of lung cancer [4,5]. The renal proximal convoluted tubules show the highest accumulation of Cd, which is one the common causes of irreversible glomerular filtration insufficiency and chronic kidney disease [6,7]. Prolonged Cd exposures have also been associated with many other health disorders such as neurodegeneration [8], bone demineralization [9], anemia [10], cardiovascular disease [11] and diabetes [12].

Molecular mechanisms by which Cd disrupts human cell physiology remain incompletely understood. Cd(II) lacks reactivity with DNA or RNA but it exhibits strong binding to Cys-SH in proteins and glutathione [13]. Conjugation of Cd(II) by the SH group of *N*-acetylcysteine is responsible for chemoprotection by this compound [14]. Binding of Cd to specific proteins was linked to oxidative stress [15,16] and inhibition of Zn-finger DNA repair proteins [17]. Protein damage by Cd in human cells triggers the formation of misfolded proteins as evidenced by the accumulation of polyubiquitinated proteins and cytosolic aggresomes [18–20]. Cd is also a well-known activator of the heat shock response driven by the sensor of misfolded proteins – the transcription factor HSF1 [21]. Damage by Cd to the hypoxia-inducible transcription factors HIF1α and HIF2α in human cells resulted in their denaturation and insolubility [22,23]. Structural damage causing protein unfolding can be highly detrimental to cell physiology due to a strong propensity of damaged proteins to act as seeds for misfolding of other proteins and formation of toxic aggregates [24,25]. In heat-shocked human cells, large proteins and proteins with long intrinsically unstable domains were the most susceptible to the loss of solubility [26,27]. It is currently unclear what groups of human proteins are particularly vulnerable to structural damage by Cd. The fate of misfolded proteins in Cd-treated human or other mammalian cells is also unknown as Cd inhibits autophagy [28,29], which normally is an important process for disposal of protein aggregates.

In this work, we investigated a global proteotoxic stress induced by mildly cytotoxic doses of Cd(II) in three human cell lines, including primary renal proximal tubule epithelial cells. We explored the origin of Cd(II)-damaged proteins and examined the role of ubiquitination and proteasomes in disposal of structurally abnormal proteins and Cd(II) tolerance by human cells.

## 2. Materials and methods

### 2.1. Cell culture

Human lung epithelial cells (H460, cat. # HTB-177), immortalized human kidney cells (293T, cat. # CRL-3216) and primary renal proximal tubule epithelial cells (RPTEC, cat. # PCS400010) were obtained from ATCC and maintained at 37 °C in 95% air and 5% CO_2_. H460 cells were grown in RPMI-1640 media (ThermoFisher, cat. #22400089) containing 10% (v/v) fetal bovine serum (FBS) and 1% penicillin/streptomycin. 293T kidney cells were cultivated in DMEM media (ThermoFisher, cat. #12430062) with 10% FBS and 1% antibiotics. Primary RPTEC cells were propagated under low serum (0.5%) conditions in a vendor-recommended media supplemented with growth factors (ATCC, cat. #PCS400030 + PCS400040).

### 2.2. Treatments of cells

Cells were seeded at approximately 60% confluence. Next day, freshly prepared CdCl_2_ (Sigma-Aldrich, cat. #239208) was added to cells for treatments alone and in the presence of specific inhibitors of ubiquitination (TAK-243, MedChemExpress, cat. #HY-100487), proteasome activity (MG132, Selleckchem, cat. #S2619), protein synthesis (cycloheximide, Sigma-Aldrich, cat. #01810) and integrated stress response (ISRIB, MedChemExpress, cat. #HY-12495A).

### 2.3. Cytotoxicity

Cellular toxicity of Cd and other treatments was evaluated using the CellTiter-Glo luminescent assay (Promega, cat. #G7571). H460, 293T and RPTEC cells were seeded at 4000 cells/well into optical bottom cell culture plates (ThermoFisher Scientific, cat. #165305) and grown overnight before the addition of CdCl_2_ and inhibitors. Assay measurements were taken at 48 h after treatments.

### 2.4. Immunoblotting

Cells were collected by trypsinization, washed twice with cold PBS and processed for cellular protein fractionation. Soluble proteins were extracted by the addition of 100 μL of a lysis buffer (0.3% Triton X-100, 20 mM HEPES pH 7.0, 100 mM NaCl) supplemented with protease/phosphatase inhibitors (ThermoFisher, cat. #PI78443). After incubation at 4 °C for 10 min with gentle shaking, soluble and insoluble cell fractions were separated by centrifugation at 10 000g for 5 min at 4 °C. A final concentration of 2% SDS was added to soluble supernatants before boiling for 10 min to inactivate ubiquitin proteases. Cell pellets were washed with 50 μL of the soluble lysis buffer, centrifuged and supernatants were discarded. Insoluble protein extracts were obtained by solubilizing the cell pellets in 100 μL of 2% SDS lysis buffer (2% SDS, 50 mM Tris-HCl pH 6.8, 10% glycerol, protease and phosphatase inhibitors, 1 mM PMSF, 5 mM EDTA) via boiling for 10 min. Proteins were separated by SDS-PAGE and transferred onto PVDF or nitrocellulose membranes using semidry transfer for small proteins and overnight wet transfer for large proteins and ubiquitin conjugates. Primary antibodies: K48-ub (Cell Signaling, 4289), p53 (Santa Cruz, sc-126), MCL1 (Cell Signaling, 5453), c-MYC (Cell Signaling, 13987), BCL-XL (Cell Signaling, 2764), BCL-2 (Cell Signaling, 2870), FK2 (Cayman, 14220), ubiquitin (Cell Signaling, 58395), phospho-eIF2α (Cell Signaling, 9721), eIF2α (Cell Signaling, 5324), ATF4 (Cell Signaling, 11815), SOD1 (Cell Signaling, 2770), fibrillarin (Cell Signaling, 2639), histone H3 (Cell Signaling, 4499), HK2 (Cell Signaling, 2106), RRM1 (Cell Signaling, 3388), XPA (Santa Cruz, sc-56813). Horseradish peroxidase-conjugated goat anti-mouse (#7076) and goat anti-rabbit (#7074) secondary antibodies were obtained from Cell Signaling.

### 2.5. Microscopy

H460 cells were seeded on round coverslips (Corning, cat. #354088) and next day treated with CdCl_2_ alone and in the presence of cycloheximide. Cells twice washed with PBS, were fixed with 3.7% paraformaldehyde (Sigma, cat. #47608) for 15 min at room temperature and refrigerated in PBS overnight. Following cell permeabilization by addition of 0.5% Triton X-100 for 15 min at room temperature, non-specific antibody binding was blocked by incubation with 2% FBS for 30 min at 37 °C. Immunostaining of polyubiquitinated proteins was performed using FK2 antibody (1/200, 2 h, 37 °C) and secondary antibody goat anti-mouse IgG Ax647 (ThermoFisher, A21235, 1/200 for 1 h at room temperature in the dark). Aggresomes of misfolded proteins were detected by staining with the ProteoStat dye (Enzo, cat. #51035). Nuclei were counterstained with Hoechst 33342. Coverslips were mounted on slides (VWR, cat. #48311–703) using Cytoseal mounting medium (Epredia, cat. #8310–4). Confocal images were obtained on Zeiss LSM 880 confocal microscope using oil 63× magnification.

### 2.6. Cellular uptake of cadmium

Cellular levels of Cd were measured by inductively coupled mass spectrometry (Thermo Scientific X-series-2 ICP-MS) using a previously described procedure [20].

### 2.7. Labeling of old and new proteins

The methionine analogue L-azidohomoalanine (AHA, ChemScene, cat. #0120777) was used to label proteins during translation. Cells were incubated with a medium containing 1 mM AHA for 3 h to label long-lived (“old” during Cd incubations) proteins. After washing with PBS, cells were chased for 2 h with 2 mM L-methionine (Sigma, cat. #M9625) and washed again before incubations with Cd for 4 h. To label nascent proteome under metal exposure, 1 mM AHA was added during the last hour of Cd treatments (“new” proteins). Cells were then collected by trypsinization and processed for protein extractions as described for immunoblotting. Cell pellets were resuspended in 50 μL of buffer (50 mM Tris pH 8.0 and 100 mM NaCl) and insoluble AHA-labeled proteins were covalently coupled to an Alexa Fluor488 alkyne (ThermoFisher, cat. #A10267) using a click-chemistry tagging kit (ThermoFisher, cat. #C10425). After centrifugation at 5000*g* for 5 min at 4°C, pellets were washed twice with 50 mM Tris-HCl, pH 8.0 and solubilized in 100 μL of 1% SDS lysis buffer (1% SDS, 50 mM Tris-HCl pH 6.8, protease and phosphatase inhibitors, 1 mM PMSF, 5 mM EDTA) by boiling for 10 min.

### 2.8. Pulldown of ubiquitinated proteins

AlexaFuor488-labeled protein samples were diluted to 0.2% SDS with 50 mM Tris-HCl (pH 8.0), 1% NP-40 and incubated overnight with the ubiquitin-binding affinity beads (Cytoskeleton, cat. #UBA01B-beads). Beads were washed twice with the cold dilution buffer and one time with cold 50 mM Tris-HCl (pH 8.0). The beads were incubated with 40 μL of 2x Laemmli buffer and 2-mercaptoethanol at 99°C for 10 min followed by centrifugation at 10 000g for 5 min at 4°C. The supernatants containing ubiquitinated proteins were resolved by SDS-PAGE and the fluorescent signal of AHA-labeled proteins was detected using the Li-Cor Odyssey M imaging system (Ax488, green). Parallel gels were run for detection of ubiquitinated proteins by immunoblotting.

### 2.9. Statistics

Two-tailed, unpaired *t*-test was used to evaluate the differences between two groups. In multiple comparisons, one-way ANOVA p-values were adjusted using the Bonferroni correction.

## 3. Results

### 3.1. Formation of denatured proteins and the role of ubiquitin-proteasome system (UPS) in Cd(II) tolerance

We investigated global proteotoxic effects of Cd(II) in three human cell lines from two main target tissues: lung and kidney. Specifically, we used H460 lung epithelial cells, immortalized 293T kidney and primary kidney (RPTEC) cells derived from the proximal tubule which is the primary site of kidney damage by Cd(II) [6,7]. Proteotoxic responses in H460 cells were similar to those in normal immortalized lung cells after treatments with metals [20] and formaldehyde [30]. H460 express a constitutively active NRF2 (due to its mutationally inactivated E3 ubiquitin ligase KEAP1) and thereby provides a cellular model with the stable activity of the main electrophilic stress response for experiments utilizing inhibitors of the ubiquitin-proteasome system (UPS) and protein translation. 293T cells contain constitutively high levels of heat shock proteins, which allows for testing of the same inhibitors without perturbations in the main defense system against protein misfolding and aggregation.

**Fig. 1.**
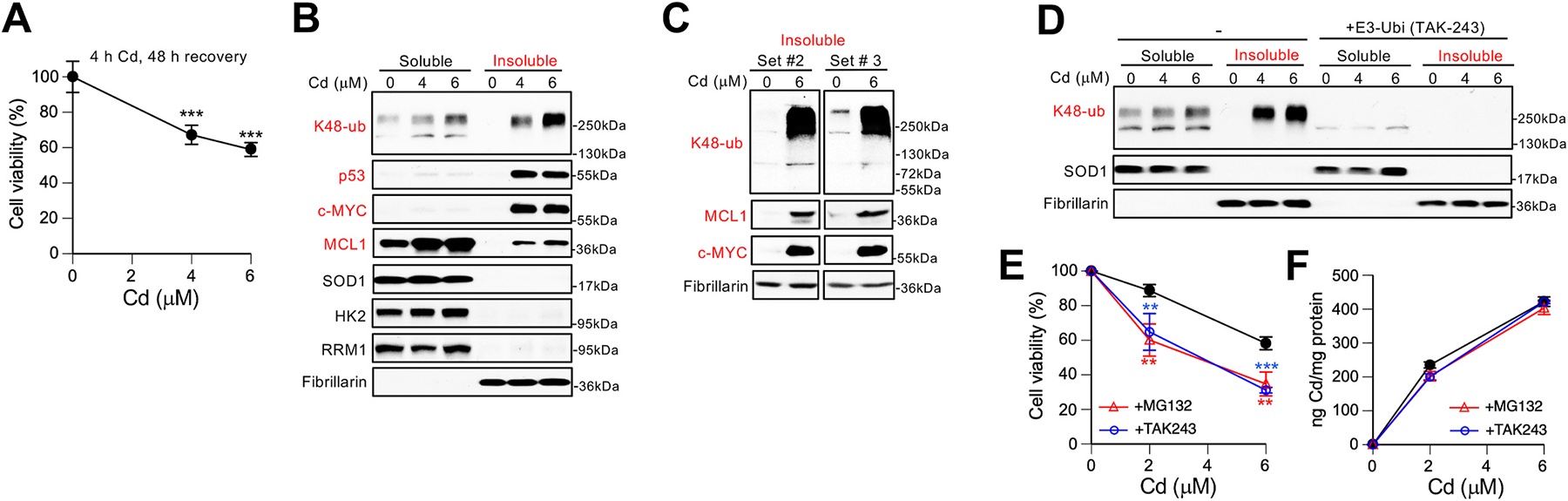
Protein polyubiquitination and the role of proteosomes in Cd tolerance by human lung cells. All Cd treatments were for 4 h and viability measurements were taken after 48 h recovery in the regular media. **(A)** Cytotoxicity of Cd treatments in H460 cells. Data are means ± SD, ***-p<0.001 relative to untreated cells, n = 4. **(B)** Cd(II)-induced changes in K48-polyubiquitination and solubility of individual proteins. SOD1 and fibrillarin were used as loading controls for soluble and insoluble fractions, respectively. **(C)** Immunoblots of insoluble proteins in two other sets of H460 samples. **(D)** Immunoblots of protein extracts from H460 cells treated with Cd alone and in the presence of the ubiquitination inhibitor TAK-243 (10 μM). **(E)** Cytotoxicity of Cd treatments in the presence of inhibitors of ubiquitination (TAK-243, 1 μM) or proteasomes (MG132, 10 μM). Data are means ± SD, **- p < 0.01, ***-p<0.001 relative to Cd alone, n = 4. **(F)** Uptake of Cd by H460 cells treated with Cd alone and in the presence of MG132 or TAK-243 as in panel E. Data are means ± SD, n = 3. In most cases, error bars were smaller than symbols.

We initially focused on cellular responses to short-term treatments with Cd to identify primary targets and to avoid mechanistic complexity arising from secondary and tertiary effects in prolonged exposures. H460 lung cells were treated with 4 and 6 μM Cd for 4 h which corresponded to approximately IC_30_ and IC_40_ doses following 48 h recovery (Fig. 1A). Induction of severe protein damage was monitored by the appearance of K48-polyubiquitinated proteins and insolubility of the normally soluble proteins. K48-linked polyubiquitin is the main proteasome-targeting protein modification [31,32] whereas protein insolubility is a well-established marker of protein denaturation *in vitro* and in cells [27,33,34]. Immunoblotting of protein extracts from Cd-treated cells showed large amounts of insoluble proteins conjugated with K48-linked polyubiquitin (Fig. 1B). Among six tested soluble proteins, antiapoptotic MCL1 and transcription factors p53 (TP53) and c-MYC were made insoluble/denatured by Cd(II). Antioxidant SOD1, glycolytic HK2 and the ribonucleotide subunit RRM1 remained soluble in Cd-treated cells. HK2 (102 kDa) and RRM1 (95 kDa) are much larger than p53 (53 kDa), c-MYC (62 kDa) or MCL1 (37 kDa) indicating that unlike thermal stress [26,27], the size of protein does not appear to be predictive of its susceptibility to denaturation by Cd(II). Extensive formation of insoluble MCL1, c-MYC and K48-polyubiquitinated proteins in Cd-treated cells was confirmed in two more independent cell extracts (Fig. 1C). Cotreatments of cells with Cd(II) and the ubiquitination inhibitor TAK-243 eliminated the presence of polyubiquitinated bands, confirming the specificity of the employed antibodies (Fig. 1D). To determine the role of functional UPS in protection against Cd toxicity, we treated cells for 4 h with Cd and nontoxic doses of inhibitors of ubiquitination (1 μM TAK-243; 114 ± 7% viability, n = 4) or proteasomal activity (10 μM MG132; 91.3 ± 5% viability, n = 4). Both inhibitors strongly increased Cd cytotoxicity (Fig. 1E), indicating that a removal of damaged proteins by UPS is a major defense mechanism against Cd in H460 cells. Uptake studies showed that MG132 and TAK-243 did not alter cellular accumulation of Cd (Fig. 1F).

**Fig. 2.**
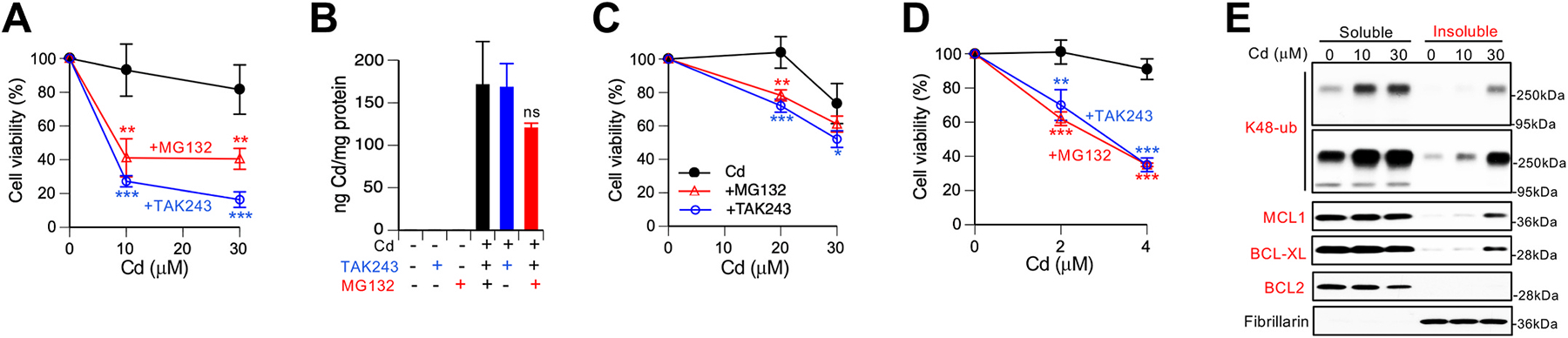
Ubiquitination and proteasome activity in Cd tolerance by 293T human kidney cells. **(A)** Cytotoxicity of 6 h Cd treatments alone and in the presence of ubiquitination (TAK-243, 0.5 μM) or proteasome (MG132, 10 μM) inhibitors. Cell viability was measured after 48 h recovery in the regular media. Means ± SD, **-p<0.01, ***-p<0.001 relative to Cd alone, n = 4. **(B)** Cellular levels of Cd after 6 h exposure to 30 μM Cd(II) alone and in the presence of TAK-243 or MG1232 as in panel B (ns – not significant, n = 3). **(C)** The impact of post-Cd addition of TAK-243 (0.1 μM) or MG132 (1 μM) for 18 h on cell viability (Cd exposures: 6 h). Cell viability was measured after 48 h recovery in the regular media. Means ± SD, *-p < 0.05, **-p < 0.01, ***-p<0.001 relative to Cd alone (n = 4). **(D)** Viability of cells cotreated with Cd and TAK-243 (0.1 μM) or MG132 (3 μM) for 18 h followed by 48 h recovery in the regular media. Means ± SD, **-p<0.01, ***-p<0.001 relative to Cd alone (n = 4). **(E)** Immunoblots of soluble and insoluble fractions of 293T cells treated with Cd for 6 h. Short and long exposures for K48-linked polyubiquitin are shown. Fibrillarin is a marker of the insoluble fraction.

293T kidney cells have a less efficient uptake of Cd than H460 and their treatments for 6 h with 10 or 30 μM Cd did not induce significant effects on cell viability. The addition of the ubiquitination or proteasome inhibitors during the same Cd treatments caused severe losses of cell viability (Fig. 2A). These striking potentiating effects on cytotoxicity were not associated with any significant changes in Cd accumulation by 293T cells (Fig. 2B). Next, we tested whether the addition of UPS inhibitors after Cd treatments could also impact cell viability, which would signify ongoing generation of damaged proteins by Cd. The ubiquitination and proteasome inhibitors were added for 18 h after the 6-h long Cd treatments, which excludes potential effects on Cd uptake. This inhibition of the UPS activity during post-Cd recovery exerted less dramatic potentiating effects than in cotreatment experiments but increases in cytotoxicity were significant for both inhibitors (Fig. 2C). To test the role of UPS in Cd toxicity at lower doses in subchronic exposures, we treated 293T cells for 18 h with 2 and 4 μM Cd, which were noncytotoxic alone (Fig. 2D). Again, the addition of ubiquitination and proteasome inhibitors to Cd treatments resulted in severe sensitization of cells to Cd toxicity. The employed doses of UPS inhibitors were only mildly cytotoxic by themselves (70.5 ± 2.3% viability for 3 μM MG132 and 73.1 ± 5.5% for 0.1 μM TAK-243, n = 4). Confirming the activation of UPS in Cd-treated 293T cells, immunoblotting with antibodies for K48-linked polyubiquitin showed accumulation of this proteolytic modification in both soluble and insoluble protein fractions (Fig. 2E). Cd also caused severe damage to two major antiapoptotic proteins MCL1 and BCL-XL, as evidenced by their loss of solubility which is indicative of protein denaturation. BCL2, another antiapoptotic protein from the same family, did not show a loss of solubility although the abundance of its soluble form was noticeably lower at the high Cd dose, potentially reflecting a more efficient disposal of Cd-damaged BCL2 relative to those of MCL1 or BCL-XL.

**Fig. 3.**
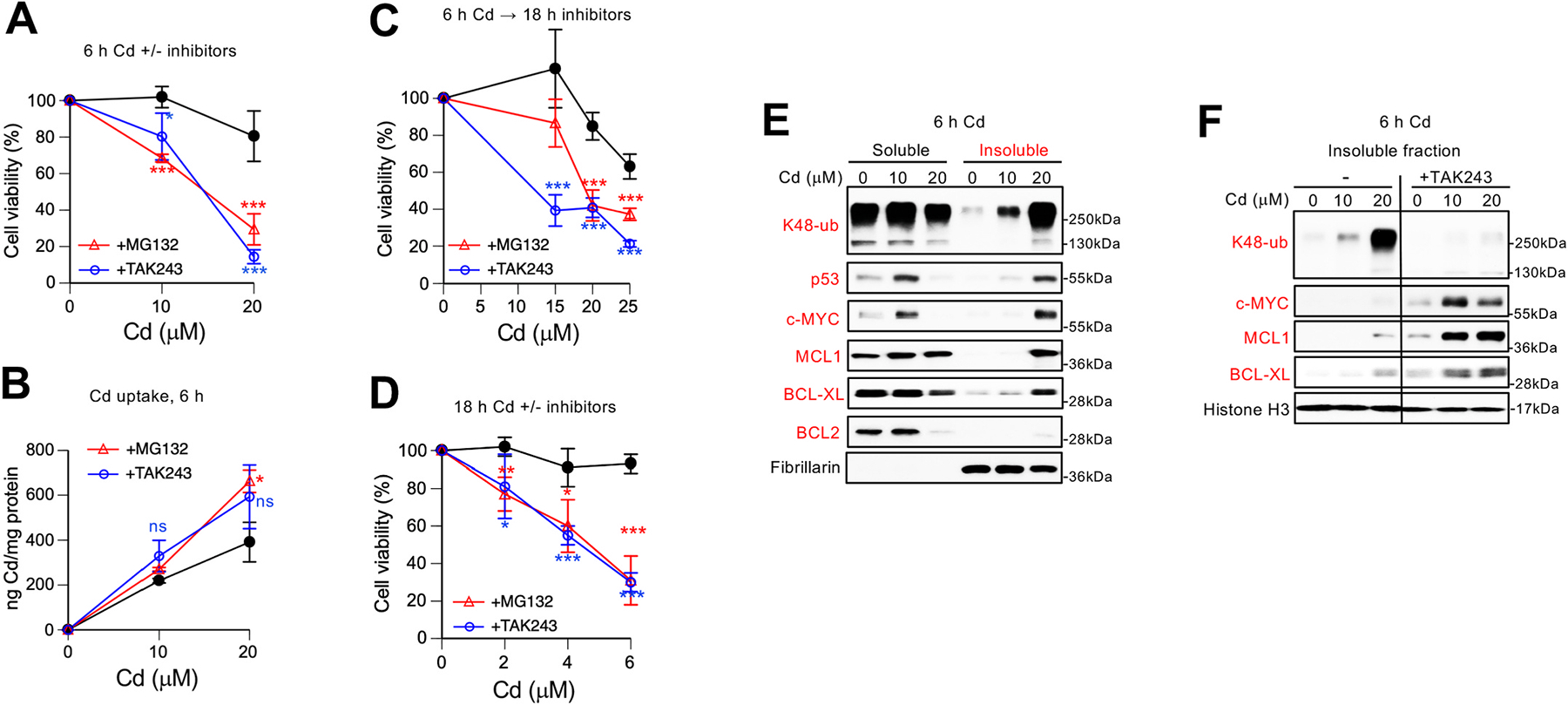
Role of ubiquitination and proteasomes in Cd resistance of primary human renal proximal tubule epithelial cells (RPTEC). All data in graphs are means ± SD. Statistics: *-p< 0.05, **-p< 0.01, ***-p< 0.001. **(A)** Cytotoxicity of Cd treatments (6 h) in the presence of TAK-243 (0.5 μM) or MG132 (5 μM). Cell viability was assessed after 48 h recovery in the regular medium (n = 4). **(B)** Cd uptake by cells treated as in panel A (n = 3). **(C)** Viability of cells treated with Cd for 6 h followed by 18 h incubation with TAK-243 (0.1 μM) or MG132 (3 μM) and then 48 h recovery in the regular media (n = 4). **(D)** Cytotoxicity of 18 h Cd treatments in the presence of TAK-243 (0.1 μM) or MG132 (3 μM) followed by 48 h recovery (n = 4). **(E)** Immunoblots of soluble and insoluble proteins from cells treated with Cd for 6 h. Fibrillarin was used as a marker of the insoluble fraction. **(F)** Inhibition of ubiquitination (0.5 μM TAK-243) led to hyperaccumulation of insoluble proteins in Cd-treated primary kidney cells. Images for TAK243-treated and untreated cells are from the same membranes with the same exposures after cropping out unrelated intervening lanes.

Similarly to immortalized cells, treatments of primary human kidney cells (RPTEC) for 6 h in the presence of the UPS inhibitors TAK-243 and MG132 resulted in very high levels of toxicity by otherwise nontoxic concentrations of Cd (Fig. 3A). Cd accumulation by RPTEC cells was unaffected by these inhibitors at the lower 10 μM Cd concentration and elevated by MG132 but not TAK-243 for 20 μM Cd exposures (Fig. 3B). Thus, potentiating effects of UPS inhibitors on Cd toxicity in primary cells under cotreatment conditions were largely metal uptake-independent. The addition of both UPS inhibitors for 18 h after the completion of metal treatments, which excludes any uptake effects, also strongly enhanced cytotoxicity of Cd (Fig. 3C). Finally, we measured viability of RPTEC cells treated for 18 h with low, normally noncytotoxic concentrations of Cd and again found that inhibition of UPS activity led to a potent enhancement of Cd toxicity at all tested doses (Fig. 3D). Immunoblotting of protein extracts from Cd-treated RPTEC cells confirmed activation of UPS as evidenced by a large accumulation of insoluble proteins with proteolytic K48-linked polyubiquitin (Fig. 3E). Similar to findings in 293T cells, Cd also induced damage to the main antiapoptotic proteins in primary kidney cells, which manifested in the formation of insoluble/denatured MCL1 and BCL-XL and the loss of BCL2. Primary kidney cells further showed a severe injury by Cd to two short-lived transcription factors p53 and c-MYC, resulting in their insolubility (Fig. 3E). Addition of TAK-243 dramatically increased the amounts of insoluble c-MYC, MCL1 and BCL-XL by Cd (Fig. 3F), confirming a critical role of ubiquitination-dependent proteolysis in disposal of Cd-denatured proteins and cytoprotection. Thus, our studies in three human cell lines found consistent evidence for a major role of global protein damage in Cd toxicity and showed a critical importance of protein polyubiquitination and proteasome activity for Cd tolerance.

### 3.2. Vulnerability of newly synthesized proteins to damage by Cd

**Fig. 4.**
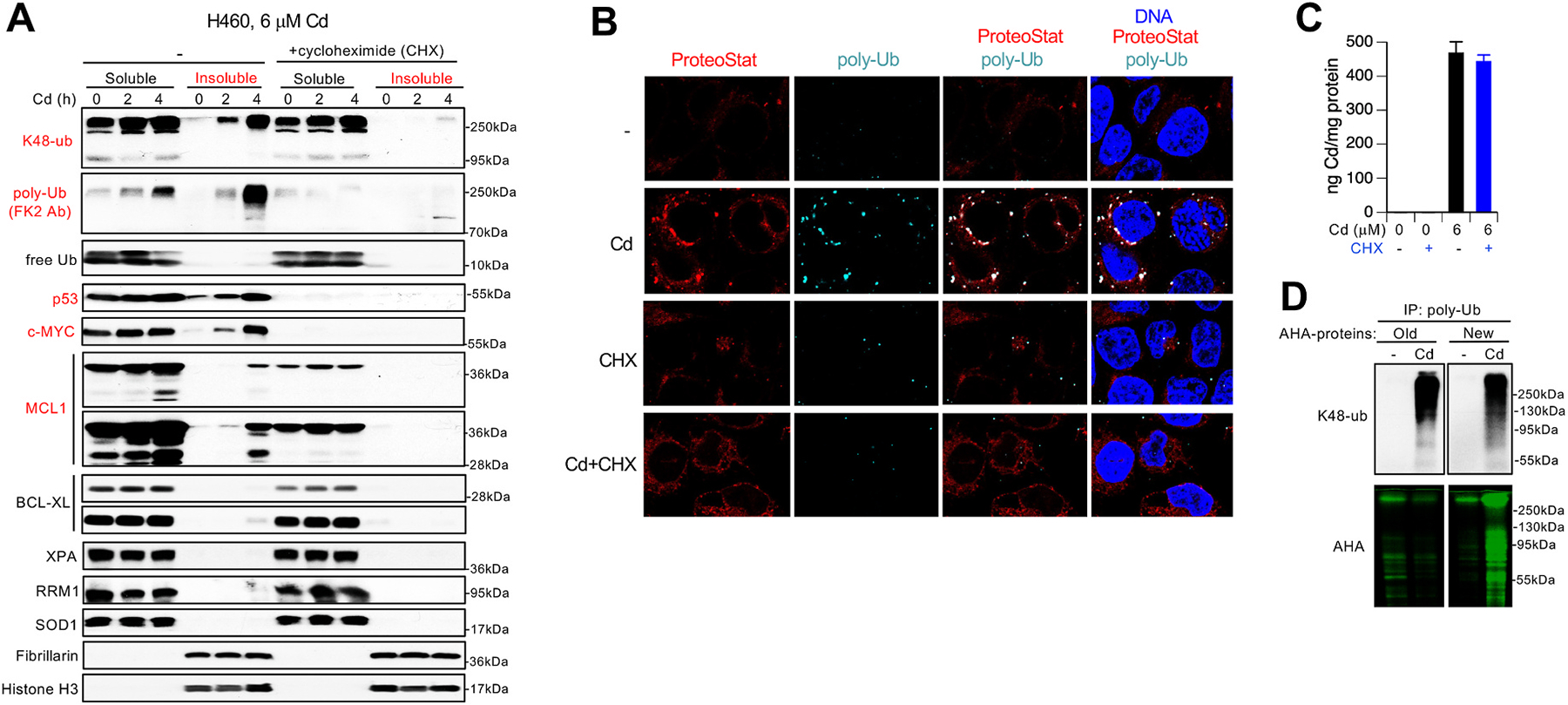
Newly synthesized proteins are targets of Cd proteotoxicity in human lung cells. **(A)** Immunoblots of soluble and insoluble extracts from control and Cd-treated H460 cells in the absence and presence of cycloheximide (CHX, 100 μg/mL). **(B)** Confocal images of aggresomes and polyubiquitin in H460 cells treated for 4 h with 6 μM Cd alone and in the presence of 100 μg/mL CHX. Cells were costained with FK2 antibody (polyubiquitin-specific), the aggresome-binding dye ProteoStat and the DNA dye Hoechst. **(C)** Cd accumulation in H460 cells treated for 4 h with 6 μM Cd alone and in the presence 100 μg/mL CHX. Data are means ± SD, n = 3. **(D)** Detection of AHA-labeled old and new proteins in the polyubiquitin-immunoprecipitates from control and Cd-treated cells (6 μM, 4 h).

The observed susceptibility of high-turnover proteins (MCL1, p53 and c-MYC) to Cd-induced denaturation raised a possibility that newly translated proteins could be particularly vulnerable to Cd damage. To test this hypothesis, we examined the formation of damaged proteins by Cd in the presence of the protein synthesis inhibitor cycloheximide (CHX). H460 cells, which showed the most rapid appearance of damaged proteins during Cd treatments, were examined first for cotreatments with 100 μg/mL CHX, a concentration which we previously validated as effective in this line [35]. We found that the addition of CHX completely eliminated the production of insoluble polyubiquitinated proteins by Cd, as shown by immunoblotting with antibodies recognizing K48-linked polyubiquitin or poly-linkage polyubiquitin (FK2 antibody) (Fig. 4A). The prevention of polyubiquitination by CHX in Cd-treated cells did not result from the deficiency in free ubiquitin which, consistent with its diminished utilization, was in fact restored for the higher Cd dose. As a consequence of their high turnover, p53 and c-MYC proteins were undetectable in CHX-treated cells. Cells incubated with CHX had lower levels of soluble antiapoptotic MCL1 (consistent with its high turnover) and completely lost the ability to generate insoluble MCL1 by Cd. Cd treatments for 4 h induced the accumulation of a shorter form of MCL1 which similarly to the main longer form was made insoluble by Cd in the absence of CHX. MCL1 generates two products through alternative splicing where a larger form is antiapoptotic whereas a smaller form is proapoptotic [36]. Antiapoptotic BCL-XL was relatively long-lived in H460 cells (evidenced by its modest decreases in CHX-treated cells) and showed only a marginal insolubility after Cd treatments, likely reflecting a small fraction of its nascent form. Consistent with this trend, other slow-turnover proteins (evidenced by their similar abundance in cells with CHX) SOD1, RRM1 and XPA did not undergo denaturation/insolubility by Cd(II) (Fig. 4A). The lack of denatured mature XPA is particularly notable as it contains a C4-type Zn-finger domain that apparently resistant to Cd(II) binding in its Zn-occupied form. Cd(II) binds to this XPA domain in *vitro* [17].

Excessive accumulation of misfolded proteins in cells can be detected by the presence of cytosolic aggresomes which are formed to segregate damaged proteins and prevent their toxic aggregation with normal proteins [37,38]. We found that Cd-treated H460 cells showed a large number of perinuclear aggresomes detected by staining with ProteoStat, a fluorescent probe for misfolded proteins with amyloid-like structures [39], and polyubiquitin-specific FK2 antibody (Fig. 4B). The majority of aggresomes showed a bright staining for both probes. In agreement with immunoblotting results, treatments of cells with Cd in the presence of the protein synthesis inhibitor CHX completely prevented the accumulation of polyubiquitinated proteins and eliminated the formation of ProteoStat-binding protein aggregates. Metal measurements showed that CHX did not change the ability of cells to take up Cd (Fig. 4C). To test more directly susceptibility of mature and new proteins to damage by Cd, we labeled cellular proteins with the Met analogue AHA at different times before Cd addition. Old (mature) proteins were labeled with AHA 2 h before the addition of Cd whereas newly synthesized proteins were labeled by the addition of AHA for the last hour of Cd treatments. Cellular proteins were incubated with the ubiquitin-binding affinity beads under denaturing conditions to ensure that only directly polyubiquitinated targets are isolated. Immunoblotting showed similar amounts of K48-polyubiquitinated proteins captured by the beads from cells with labeled “old” and “new” proteins (Fig. 4D). In a striking contrast, only newly synthesized proteins were detected in the polyubiquitinated fraction of Cd-treated cells demonstrating hypersensitivity of immature proteins to Cd damage.

**Fig. 5.**
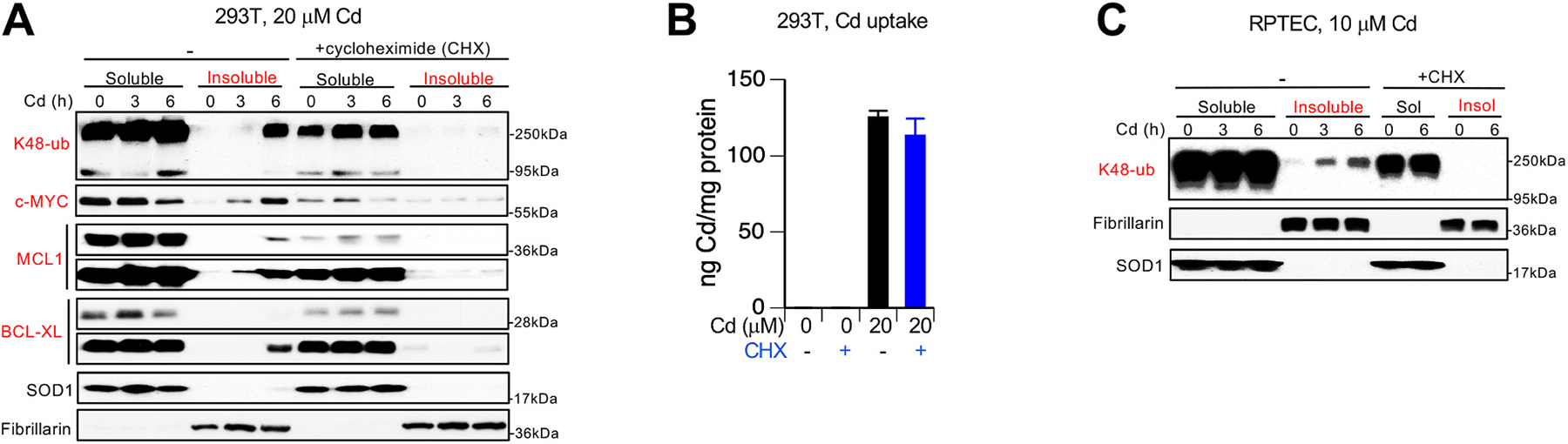
Prevention of protein ubiquitination and denaturation by inhibition of protein synthesis in human kidney cells. **(A)** Absence of insoluble polyubiquitinated and denatured proteins in 293T cells treated with Cd in the presence of the protein translation inhibitor cycloheximide (CHX, 100 μg/mL). For MCL1 and BCL-XL, shorter and longer exposures are shown. SOD1 and fibrillarin were used as markers of soluble and insoluble protein fractions, respectively. **(B)** Cd accumulation in 293T cells treated with 20 μM Cd and 100 μg/mL CHX for 6 h. Data are means ± SD, n = 3. **(C)** Absence of insoluble polyubiquitinated proteins in RPTEC cells cotreated with 10 μM Cd and 100 μg/mL CHX.

Similar to H460 cells, blockage of protein synthesis also prevented the formation of insoluble polyubiquitinated proteins and insolubility of c-MYC and antiapoptotic proteins in Cd-treated 293T kidney cells (Fig. 5A). BCL-XL was more short-lived in 293T cells (evidenced by larger decreases in the presence of CHX) and its steady-state expression should be maintained by correspondingly higher rates of synthesis. A higher fraction of nascent polypeptides in its total cellular pool can explain increased insolubility/vulnerability of BCL-XL in response to Cd. Uptake experiments showed that inhibition of protein synthesis by CHX did not affect accumulation of Cd by 293T cells (Fig. 5B). The importance of active translation for the formation of Cd-damaged proteins undergoing proteolytic polyubiquitination was also found in primary kidney cells (Fig. 5C). Overall, these studies showed that newly synthesized proteins were the main target for Cd-induced proteotoxicity manifested in the formation of polyubiquitinated proteins, formation of cytosolic aggresomes and insolubility/denaturation of proteins with high turnover rates.

### 3.3. Integrated stress response (ISR) in protection against Cd proteotoxicity

**Fig. 6.**
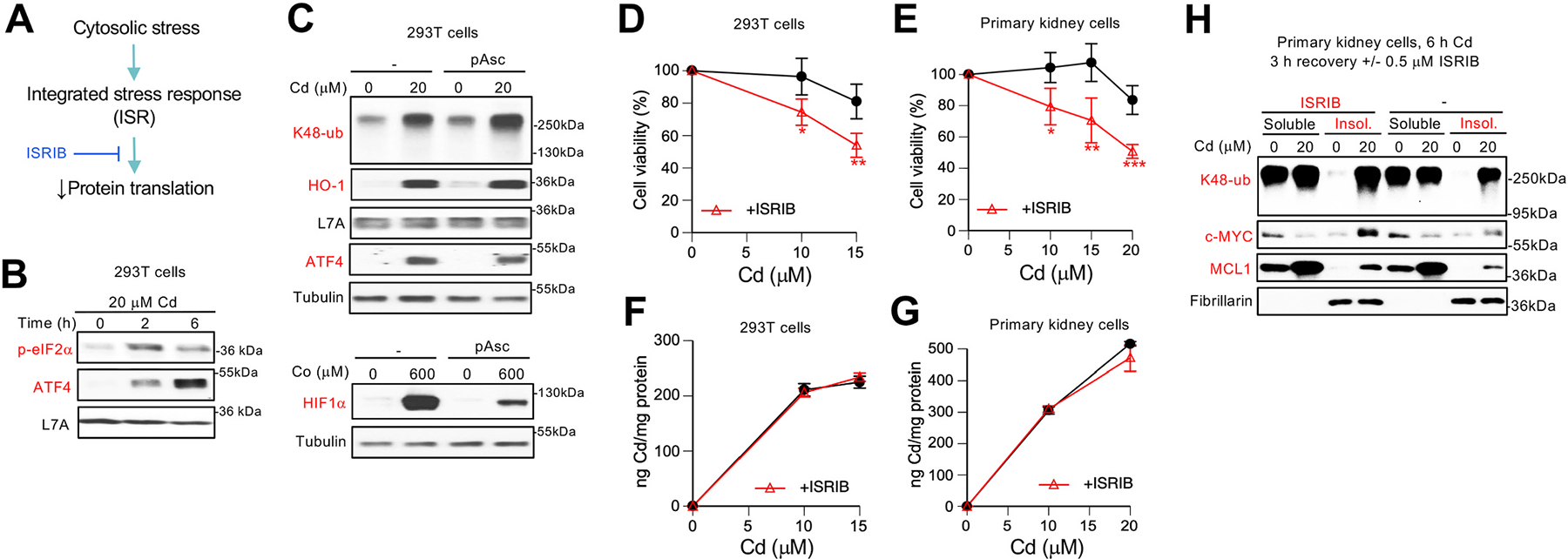
Role of integrated stress response (ISR) in protection against Cd proteotoxicity in human kidney cells. **(A)** Schematic representation of Integrated Stress Response (ISR). Cellular cytosolic stress triggers ISR activation that leads to inhibition of protein translation. The ISR inhibitor ISRIB prevents downregulation of protein synthesis under stress. **(B)** Immunoblots for markers of ISR activation phospho-eIF2α and ATF4 in Cd-treated 293T cells. **(C)** Proteotoxic stress in 293T cells preincubated with 400 μM ascorbate phosphate (pAsc) and then treated with for 6 h with Cd(II) (top panel) or CoCl_2_ (bottom panel). Whole protein extracts (2% SDS lysates) were used in these immunoblots. **(D)** Viability of 293T and **(E)** primary RPTEC cells treated with Cd for 6 h alone and in the presence of ISRIB (100 nM) followed by 48 h recovery. ISRIB was also present during the post-Cd recovery. Means ± SD, *-p <0.05, **-p<0.01 (n = 4). **(F)** Cd uptake by 293T and **(G)** primary RPTEC cells treated with Cd and ISRIB as in panels D and E. Means ± SD, n = 3. **(H)** Immunoblots of soluble and insoluble fractions of primary RPTEC cells treated with Cd for 6 h followed by 3 h recovery without and with the addition of ISRIB (500 nM).

Many forms of cytoplasmic stress activate a common adaptive process known as the integrated stress response (ISR). ISR is initiated by the phosphorylation of the eukaryotic translation initiation factor eIF2α, which decreases the rates of global protein synthesis and also leads to the upregulation of selected proteins such as the transcription factor ATF4 [40,41] (Fig. 6A). We found that Cd treatments of 293T cells activated ISR as evidenced by an early increase in eIF2α phosphorylation followed by a strong induction of ATF4 (Fig. 6B). A diminished level of eIF2α phosphorylation at 6 h vs 2 h Cd probably reflects the activation of the negative feedback mechanism via induction of GADD34 phosphatase by ISR [40,41]. Activation of ISR (ATF4), NRF2 (HO-1) and polyubiquitination by Cd were all unaffected by the antioxidant ascorbate (added in its stable form of ascorbate phosphate) (Fig. 6C, top panel), arguing against a significant role of ROS in these responses. A control experiment showed that ascorbate-supplemented cells were resistant to the oxidant-dependent stabilization of HIF1α by redox-active Co(II) (Fig. 6C, bottom panel) [42]. Slowdown of global protein synthesis by active ISR raises a possibility that it could be protective against Cd proteotoxicity which we found to target newly translated proteins. To test the role of ISR in Cd tolerance, we examined viability of 293T and primary kidney cells treated with Cd in the presence of a well-characterized ISR inhibitor ISRIB [43,44]. Inhibition of ISR by 0.1 μM ISRIB in both cell lines significantly increased cytotoxicity of Cd at its mild doses (Fig. 6D and E). ISRIB was noncytotoxic by itself (105 ±11% viability in 293T and 109 ± 15% in RPTEC cells, n = 4). Uptake experiments in 293T and primary kidney cells showed that ISRIB did not change their accumulation of Cd (Fig. 6F and G). Immunoblotting of protein extracts from primary kidney cells treated with the ISR inhibitor after Cd removal showed increased levels of protein damage as evidenced by a higher abundance of insoluble K48-polyubiquitinated proteins and larger amounts of insoluble c-MYC and MCL1 (Fig. 6H). Thus, ISR activation is an important protective response against Cd proteotoxicity, which is consistent with its slowdown of global protein synthesis limiting the amounts of vulnerable nascent polypeptides for misfolding by Cd.

## 4. Discussion

Proteins require folding into specific conformations for the formation of catalytic centers or binding with other molecules. Disruption of the physiological folding mechanisms leads to the production of abnormal proteins with the loss of function and is associated with the formation of large clumps including the same or different proteins. These aggregates cause proteotoxic stress in cells activating protein homeostasis mechanisms to prevent pathophysiological changes or death [24,25]. Using three human cell lines, our studies here identified the main group of proteins that is hypersensitive to structural/misfolding damage by a potent toxicant Cd and determined their role in toxicity of this metal. Our findings revealed that the primary group of human proteins structurally damaged by Cd were newly synthesized polypeptides. This conclusion is supported by several lines of evidence: 1) pharmacological inhibition of protein synthesis prevented overt Cd-induced proteotoxicity such as the accumulation of proteolytic K48-polyubiquitination of proteins, loss of protein solubility and the formation of aggresomes in cells; 2) proteins with high rates of turnover (new synthesis) were especially sensitive to damage by Cd; 3) analysis of polyubiquitinated proteins from Cd-treated cells showed that they were all newly synthesized proteins; 4) inactivation of ISR, the physiological mechanism for attenuation of translation in stressed cells, enhanced the formation of damaged proteins and cytotoxicity by Cd. Previous studies in yeast found that translation inhibition blocked a large increase in cytosolic Hsp104 foci (used as a marker of protein aggregates) by Cd, however, the insoluble fraction of cells was only modestly enriched in newly synthesized proteins [45]. These findings may reflect a higher susceptibility of mature yeast proteins to misfolding relative to human proteins [26] and/or a low sensitivity of new protein analyses in the total insoluble fraction of yeast. Our studies in human cells showed that ubiquitin-dependent degradation of damaged proteins by proteasomes played a key role in protection against Cd toxicity as evidenced by a severe lethality of Cd in UPS-inhibited cells at its otherwise noncytotoxic doses. Active ISR further contributed to cellular tolerance of Cd but to a smaller degree than UPS and through a different mechanism (slower translation leading to fewer targets for Cd damage). Although inhibition of ubiquitination and proteasomes showed similar increases in Cd cytotoxicity, we cannot exclude the possibility that cells also engage ubiquitin-independent mechanisms of proteasome activation for disposal of some Cd-damaged proteins [46,47]. In addition to slower translation, some of the protective effects of ISR against Cd toxicity may also result from activities of the transcription factor ATF4 [40,41].

Mean kidney cortex concentrations of Cd in the general population from different countries varied from 19 to 82.7 μg/g wet tissue (170–738 μM Cd) [48–50], whereas in cadmium smelter workers they reached mM levels [51]. Inhalation of the mainstream tobacco smoke results in the lung deposition of CdO-containing particles ranging from <0.1 to 2 μm in diameter [52]. We calculated that internalization and dissolution of a single 0.25 μm CdO particle or a 1 μm particle with only 2% CdO in the human alveolar cell (volume = 3 pL) would deliver 173 and 222 μM Cd, respectively. Using the previously established relationship between cellular protein amounts and volume in H460 cells [53], we determined that the highest Cd concentrations in our studies delivered 169 μM Cd in H460 lung cells, 148 μM in 293T kidney cells and 180 μM in primary kidney RPTEC cells. Thus, our conclusions about toxicity and protein damage by Cd were based on findings obtained at its environmentally relevant tissue doses.

Accumulation of insoluble proteins in Cd-treated cells suggests that some damaged proteins probably are poor substrates for polyubiquitination and they are likely to form large clumps inhibiting solubilization. The transcription factor 53 was one of the proteins that became insoluble after Cd treatments. This response resembles the phenotype of structure-destabilizing cancer mutations in p53, leading to its diminished proteasomal degradation and formation of large aggregates incorporating both wild-type and mutated p53 [54]. The intrinsically unstable conformation of the DNA-binding domain of p53 is maintained by binding of a single Zn^2+^ ion to C238/C242 in L3 loop and C176/H179 in L2 loop. Zn-free DNA-binding domain of p53 is unstable and misfolds, mimicking effects of the common cancer mutations in this tumor suppressor [55,56]. Cd(II) is a strongly soft electrophile exhibiting a much higher affinity for the soft nucleophile SH-group than a moderately soft electrophile Zn(II). Thus, Cd(II) is expected to effectively outcompete Zn(II) for binding to unoccupied Cys-SH groups in the newly synthesized p53 protein, destabilizing its structure and causing misfolding/insolubility. A larger atomic size of Cd(II) and its poor ability to bind His (hard nucleophile) in proteins [57] apparently prevents the formation of a stable pseudo-physiological conformation of p53. Displacement of Zn(II) by Cd(II) in the mature p53 is expected to be slow due to stability of multiligand-bound Zn(II).

**Fig. 7.**
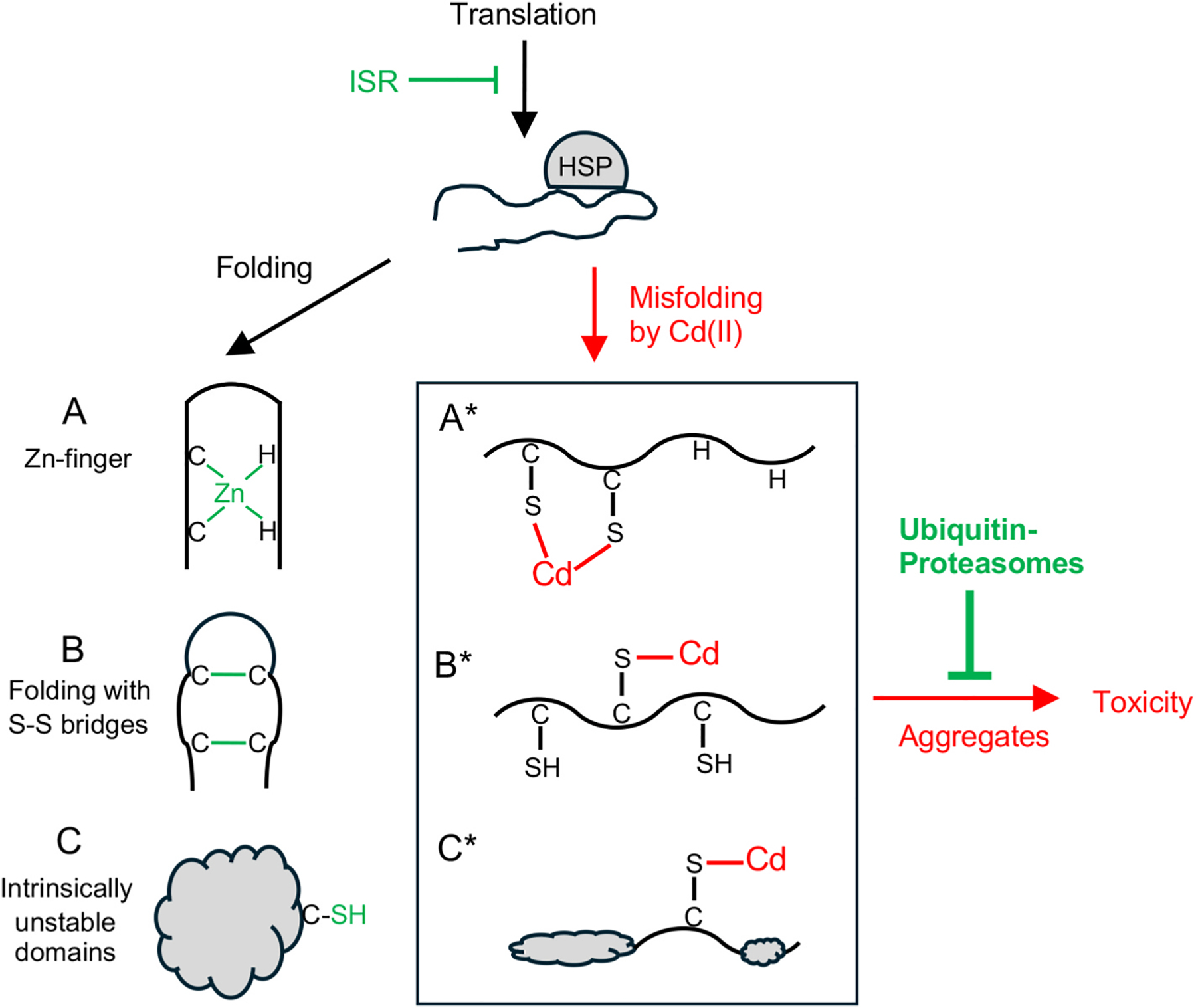
Model of Cd(II) proteotoxicity through structural damage to newly synthesized proteins. Nascent proteins are maintained in the unstructured state by bound molecular chaperons, especially by HSP70. High-affinity binding of Cd(II) to Cys-SH interferes with normal protein folding mechanisms such as the formation of Zn-fingers (C2H2-type shown), disulfide bridges or intrinsically unstable domains (Zn-stabilized and others with vulnerable Cys-SH). HSP70 and folding-assisting HSP90 contain functionally important Cys-SH groups which are targets for inactivation of these HSPs by several nucleophilic compounds [66] and could be potentially damaged by Cd(II). Protective cellular responses against Cd(II) proteotoxicity include a slowdown of translation by activated integrated stress response (ISR) and polyubiquitination and degradation of damaged proteins by proteasomes.

Independent of their ligand preferences, toxic transition metals show stable binding to proteins. However, only SH-reactive Co(II) and Cd(II) induced protein polyubiquitination in cells but not the hard electrophile Cr(III) with preferential binding to negatively charged carboxy groups [20]. We suggest that Cd(II) attachment to structurally important Cys-SH groups in newly synthesized proteins with immature structure is largely responsible for their hypersensitivity to misfolding damage (Fig. 7). Nascent polypeptides are unstructured and supported by bound molecular chaperones. Cys residues are critical for the establishment of the functional protein conformations through the disulfide-mediated protein folding and the formation of Zn(II)-stabilized structures such as Zn-finger domains. Cys-Cys disulfides are unreactive with Cd(II) whereas multiligand-bound Zn(II) in Zn-finger domains is relatively resistant for displacement, which makes mature proteins less sensitive to structural damage by Cd(II). Zn-finger proteins in humans include 1723 annotated members [58] and unoccupied Cys-SH groups in this very large class of proteins are expected to be highly susceptible to Cd(II) binding and the resulting structural abnormalities. The overall size of human Zn-binding cysteine proteome is greater than the number of Zn-finger proteins [59] and all proteins in which Zn(II) acts as a structural component (more broadly than just forming Zn-fingers) should be vulnerable to Cd(II) binding and structural damage in their nascent forms. Many members of a large group of human proteins containing intrinsically disordered domains [60,61] are also likely to exhibit a heightened vulnerability to misfolding by Cd binding. MCL1 and c-MYC, two proteins which we found to be very sensitive to the Cd-induced loss of solubility, do not have known Zn-binding sites or structure-stabilizing disulfide bonds. However, both proteins contain large intrinsically disordered regions with Cys-SH sites (C171 in c-MYC, C286 in MCL1) whose chemical modification causes disabling structural damage [62, 63]. On the total proteome level, a relative vulnerability of different classes of Cd-binding proteins to structural damage/denaturation can be further explored through application of protein mass-spectrometry [64, 65]. Although oxidation of Cys-SH groups can mimic some effects of their adduction by Cd(II), the contribution of oxidative damage to the global proteotoxicity in our mildly cytotoxic Cd treatments was probably minor if any. Cd(II) is redox-inactive and its oxidative stress in cells is indirect. Oxidized protein-SH are also repaired by effective enzymes (thioredoxin, glutaredoxin) whereas Cd binding lacks active reversal processes and is more persistent. Furthermore, we found that validated concentrations of the antioxidant ascorbate did not change protein polyubiquitination or activation of the Cys-SH damage sensor NRF2 by Cd in human lung [20] or kidney cells (this study).

## Data Availability

Data will be made available on request.

## References

[1] Thévenod F, Lee WK, Toxicology of cadmium and its damage to mammalian organs, Met. Ions. Life. Sci 11 (2013) 415–490, 10.1007/978-94-007-5179-8_14. 23430781

[2] Genchi G, Sinicropi MS, Lauria G, Carocci A, Catalano A, The effects of cadmium toxicity, Int. J. Environ. Res. Publ. Health 17 (11) (2020) 3782, 10.3390/ijerph17113782. PMC7312803 32466586

[3] Hou D, Jia X, Wang L, , Global soil pollution by toxic metals threatens agriculture and human health, Science 388 (6744) (2025) 316–321, 10.1126/science.adr5214. 40245139

[4] Nawrot T, Plusquin M, Hogervorst J, , Environmental exposure to cadmium and risk of cancer: a prospective population-based study, Lancet Oncol. 7 (2) (2006) 119–126, 10.1016/S1470-2045(06)70545-9. 16455475

[5] Nawrot TS, Martens DS, Hara A, , Association of total cancer and lung cancer with environmental exposure to cadmium: the meta-analytical evidence, Cancer Causes Control 26 (9) (2015) 1281–1288, 10.1007/s10552-015-0621-5. 26109463

[6] Johri N, Jacquillet G, Unwin R, Heavy metal poisoning: the effects of cadmium on the kidney, Biometals 23 (5) (2010) 783–792, 10.1007/s10534-010-9328-y. 20354761

[7] Jain RB, Cadmium and kidney function: concentrations, variabilities, and associations across various stages of glomerular function, Environ. Pollut 256 (2020) 113361, 10.1016/j.envpol.2019.113361. 31668955

[8] Arruebarrena MA, Hawe CT, Lee YM, Branco RC, Mechanisms of cadmium neurotoxicity, Int. J. Mol. Sci 24 (23) (2023) 16558, 10.3390/ijms242316558. 38068881 PMC10706630

[9] Kjellström T, Mechanism and epidemiology of bone effects of cadmium, IARC Sci. Publ 118 (1992) 301–310. 1303956

[10] Horiguchi H, Teranishi H, Niiya K, Aoshima K, Katoh T, Sakuragawa N, Kasuya M, Hypoproduction of erythropoietin contributes to anemia in chronic cadmium intoxication: clinical study on Itai-itai disease in Japan, Arch. Toxicol 68 (10) (1994) 632–636, 10.1007/BF03208342. 7857202

[11] Gallagher CM, Meliker JR, Blood and urine cadmium, blood pressure, and hypertension: a systematic review and meta-analysis, Environ. Health Perspect 118 (12) (2010) 1676–1684, 10.1289/ehp.1002077. 20716508 PMC3002186

[12] Buha A, Đukić-Ćosić D, Ćurčić M, , Emerging links between cadmium exposure and insulin resistance: human, animal, and cell Study data, Toxics 8 (3) (2020) 63, 10.3390/toxics8030063. 32867022 PMC7560347

[13] Yu HT, Zhen J, Leng JY, Cai L, Ji HL, Keller BB, Zinc as a countermeasure for cadmium toxicity, Acta Pharmacol. Sin 42 (3) (2021) 340–346, 10.1038/s41401-020-0396-4. 32284539 PMC8027184

[14] Luczak MW, Zhitkovich A, Role of direct reactivity with metals in chemoprotection by N-acetylcysteine against chromium(VI), cadmium(II), and cobalt(II), Free Radic. Biol. Med 65 (2013) 262–269, 10.1016/j.freeradbiomed.2013.06.028. 23792775 PMC3823631

[15] Chrestensen CA, Starke DW, Mieyal JJ, Acute cadmium exposure inactivates thioltransferase (Glutaredoxin), inhibits intracellular reduction of protein-glutathionyl-mixed disulfides, and initiates apoptosis, J. Biol. Chem 275 (34) (2000) 26556–26565, 10.1074/jbc.M004097200. 10854441

[16] Romanova N, Sule K, Issler T, , Cadmium-cardiolipin disruption of respirasome assembly and redox balance through mitochondrial membrane rigidification, J. Lipid Res 66 (3) (2025) 100750, 10.1016/j.jlr.2025.100750. 39880166 PMC11905837

[17] Hartwig A, Zinc finger proteins as potential targets for toxic metal ions: differential effects on structure and function, Antioxidants Redox Signal. 3 (4) (2001) 625–634, 10.1089/15230860152542970. 11554449

[18] Song C, Xiao Z, Nagashima K, , The heavy metal cadmium induces valosin-containing protein (VCP)-mediated aggresome formation, Toxicol. Appl. Pharmacol 228 (3) (2008) 351–363, 10.1016/j.taap.2007.12.026. 18261755 PMC2692476

[19] Fujiki K, Tanabe K, Suzuki S, , Blockage of Akt activation suppresses cadmium-induced renal tubular cellular damages through aggrephagy in HK-2 cells, Sci. Rep 14 (2024) 14552, 10.1038/s41598-024-64579-3. 38914593 PMC11196260

[20] Ursu GM, Krawic C, Zhitkovich A, Nuclear SUMOylation and proteotoxic stress responses to metals with different ligand preferences, Chem. Res. Toxicol 38 (5) (2025) 942–953, 10.1021/acs.chemrestox.5c00040. 40243484 PMC12308312

[21] Anckar J, Sistonen L, Regulation of HSF1 function in the heat stress response: implications in aging and disease, Annu. Rev. Biochem 80 (2011) 1089–1115, 10.1146/annurev-biochem-060809-095203. 21417720

[22] Meyers LM, Krawic C, Luczak MW, Zhitkovich A, Vulnerability of HIF1α and HIF2α to damage by proteotoxic stressors, Toxicol. Appl. Pharmacol 445 (2022) 116041, 10.1016/j.taap.2022.116041. 35504338 PMC9334845

[23] Schreiber T, Scharner B, Thévenod F, Insoluble HIFa protein aggregates by cadmium disrupt hypoxia-prolyl hydroxylase (PHD)-hypoxia inducible factor (HIFa) signaling in renal epithelial (NRK-52E) and interstitial (FAIK3–5) cells, Biometals 37 (6) (2024) 1629–1642, 10.1007/s10534-024-00631-z. 39256317 PMC11618182

[24] Vendruscolo M, Proteome folding and aggregation, Curr. Opin. Struct. Biol 22 (2) (2012) 138–143, 10.1016/j.sbi.2012.01.005. 22317916

[25] Louros N, Schymkowitz J, Rousseau F, Mechanisms and pathology of protein misfolding and aggregation, Nat. Rev. Mol. Cell Biol 24 (12) (2023) 912–933, 10.1038/s41580-023-00647-2. 37684425

[26] Leuenberger P, Ganscha S, Kahraman A, , Cell-wide analysis of protein thermal unfolding reveals determinants of thermostability, Science. 355 (6327) (2017) eaai7825, 10.1126/science.aai7825. 28232526

[27] Sui X, Pires DEV, Ormsby AR, , Widespread remodeling of proteome solubility in response to different protein homeostasis stresses, Proc. Natl. Acad. Sci. USA 117 (5) (2020) 2422–2431, 10.1073/pnas.1912897117. 31964829 PMC7007570

[28] Lee WK, Probst S, Santoyo-Sánchez MP, , Initial autophagic protection switches to disruption of autophagic flux by lysosomal instability during cadmium stress accrual in renal NRK-52E cells, Arch. Toxicol 91 (10) (2017) 3225–3245, 10.1007/s00204-017-1942-9. 28321485

[29] Liu F, Wang XY, Zhou XP, , Cadmium disrupts autophagic flux by inhibiting cytosolic Ca^2+^-dependent autophagosome-lysosome fusion in primary rat proximal tubular cells, Toxicology 383 (2017) 13–23, 10.1016/j.tox.2017.03.016. 28347754

[30] Ortega-Atienza S, Rubis B, McCarthy C, Zhitkovich A, Formaldehyde is a potent proteotoxic stressor causing rapid heat shock transcription factor 1 activation and Lys48-Linked polyubiquitination of proteins, Am. J. Pathol 186 (11) (2016) 2857–2868, 10.1016/j.ajpath.2016.06.022. 27639166 PMC5222959

[31] Ikeda F, Crosetto N, Dikic I, What determines the specificity and outcomes of ubiquitin signaling? Cell 143 (5) (2010) 677–681, 10.1016/j.cell.2010.10.026. 21111228

[32] Swatek KN, Komander D, Ubiquitin modifications, Cell Res. 26 (4) (2016) 399–422, 10.1038/cr.2016.39. 27012465 PMC4822133

[33] Sridharan S, Kurzawa N, Werner T, , Proteome-wide solubility and thermal stability profiling reveals distinct regulatory roles for ATP, Nat. Commun 10 (2019) 1155, 10.1038/s41467-019-09107-y. 30858367 PMC6411743

[34] Mateus A, Kurzawa N, Becher I, , Thermal proteome profiling for interrogating protein interactions, Mol. Syst. Biol 16 (2020) MSB199232, 10.15252/msb.20199232. PMC7057112 32133759

[35] Wong VC, Cash HL, Morse JL, Lu S, Zhitkovich A, S-phase sensing of DNA-protein crosslinks triggers TopBP1-independent ATR activation and p53-mediated cell death by formaldehyde, Cell Cycle 11 (13) (2012) 2526–2537, 10.4161/cc.20905. 22722496 PMC3404879

[36] Bae J, Leo CP, Hsu SY, Hsueh AJ, MCL-1S, a splicing variant of the antiapoptotic BCL-2 family member MCL-1, encodes a proapoptotic protein possessing only the BH3 domain, J. Biol. Chem 275 (33) (2000) 25255–25261, 10.1074/jbc.M909826199. 10837489

[37] Kopito RR, Aggresomes, inclusion bodies and protein aggregation, Trends Cell Biol. 10 (12) (2000) 524–530, 10.1016/s0962-8924(00)01852-3. 11121744

[38] Kawaguchi Y, Kovacs JJ, McLaurin A, Vance JM, Ito A, Yao TP, The deacetylase HDAC6 regulates aggresome formation and cell viability in response to misfolded protein stress, Cell 115 (6) (2003) 727–738, 10.1016/s0092-8674(03)00939-5. 14675537

[39] Shen D, Coleman J, Chan E, , Novel cell- and tissue-based assays for detecting misfolded and aggregated protein accumulation within aggresomes and inclusion bodies, Cell Biochem. Biophys 60 (3) (2011) 173–185, 10.1007/s12013-010-9138-4. 21132543 PMC3112480

[40] Pakos-Zebrucka K, Koryga I, Mnich K, , The integrated stress response, EMBO Rep. 17 (2016) 1374–1395, 10.15252/embr.201642195. 27629041 PMC5048378

[41] Costa-Mattioli M, Walter P, The integrated stress response: from mechanism to disease, Science 368 (6489) (2020) eaat5314, 10.1126/science.aat5314. 32327570 PMC8997189

[42] Salnikow K, Donald SP, Bruick RK, Zhitkovich A, Phang JM, Kasprzak KS, Depletion of intracellular ascorbate by the carcinogenic metals nickel and cobalt results in the induction of hypoxic stress, J. Biol. Chem 279 (39) (2004) 40337–40344, 10.1074/jbc.M403057200. 15271983

[43] Sidrauski C, McGeachy AM, Ingolia NT, Walter P, The small molecule ISRIB reverses the effects of eIF2α phosphorylation on translation and stress granule assembly, eLife 4 (2015) e05033, 10.7554/eLife.05033. 25719440 PMC4341466

[44] Zyryanova AF, Weis F, Faille A, , Binding of ISRIB reveals a regulatory site in the nucleotide exchange factor eIF2B, Science 359 (6383) (2018) 1533–1536, 10.1126/science.aar5129. 29599245 PMC5889100

[45] Jacobson T, Priya S, Sharma SK, , Cadmium causes misfolding and aggregation of cytosolic proteins in yeast, Mol. Cell Biol 37 (17) (2017) e00490–16, 10.1128/MCB.00490-16. 28606932 PMC5559669

[46] Ortega-Atienza S, Krawic C, Watts L, McCarthy C, Luczak MW, Zhitkovich A, 20S immunoproteasomes remove formaldehyde-damaged cytoplasmic proteins suppressing caspase-independent cell death, Sci. Rep 7 (1) (2017) 654, 10.1038/s41598-017-00757-w. 28381880 PMC5429636

[47] Gu X, Nardone C, Kamitaki N, Mao A, Elledge SJ, Greenberg ME, The midnolin-proteasome pathway catches proteins for ubiquitination-independent degradation, Science 381 (6660) (2023) eadh5021, 10.1126/science.adh5021. 37616343 PMC10617673

[48] Thürauf J, Schaller KH, Valentin H, Weltle D, Grote K, Schellmann B, Cadmium concentrations in autopsy material from differently polluted areas of West Germany (FRG), Zentralbl Bakteriol Mikrobiol Hyg B Umwelthyg Krankenhaushyg Arbeitshyg Prav Med. 182 (4) (1986) 337–347. 3096015

[49] Bem EM, Kaszper BW, Orłowski C, Piotrowski JK, Wójcik G, Zołnowska E, Cadmium, zinc, copper and metallothionein levels in the kidney and liver of humans from central Poland, Environ. Monit. Assess 25 (1) (1993) 1–13, 10.1007/BF00549788. 24227452

[50] Hayashi C, Koizumi N, Nishio H, Koizumi N, Ikeda M, Cadmium and other metal levels in autopsy samples from a cadmium-polluted area and non-polluted control areas in Japan, Biol. Trace Elem. Res 145 (1) (2012) 10–22, 10.1007/s12011-011-9155-1. 21809055

[51] Ellis KJ, Morgan WD, Zanzi I, Yasumura S, Vartsky D, Cohn SH, Critical concentrations of cadmium in human renal cortex: dose-effect studies in cadmium smelter workers, J. Toxicol. Environ. Health 7 (5) (1981) 691–703, 10.1080/15287398109530012. 7021865

[52] Wang H, Li X, Guo J, , Distribution of toxic chemicals in particles of various sizes from mainstream cigarette smoke, Inhal. Toxicol 28 (2) (2016) 89–94, 10.3109/08958378.2016.1140851. 26865272

[53] Reynolds M, Armknecht S, Johnston T, Zhitkovich A, Undetectable role of oxidative DNA damage in cell cycle, cytotoxic and clastogenic effects of Cr(VI) in human lung cells with restored ascorbate levels, Mutagenesis 27 (4) (2012) 437–443, 10.1093/mutage/ger095. 22241526 PMC3382305

[54] Xu J, Reumers J, Couceiro JR, , Gain of function of mutant p53 by coaggregation with multiple tumor suppressors, Nat. Chem. Biol 7 (5) (2011) 285–295, 10.1038/nchembio.546. 21445056

[55] Cho Y, Gorina S, Jeffrey PD, Pavletich NP, Crystal structure of a p53 tumor suppressor-DNA complex: understanding tumorigenic mutations, Science 265 (5170) (1994) 346–355, 10.1126/science.8023157. 8023157

[56] Ha JH, Prela O, Carpizo DR, Loh SN, p53 and zinc: a malleable relationship, Front. Mol. Biosci 9 (2022) 895887, 10.3389/fmolb.2022.895887. 35495631 PMC9043292

[57] Perinelli M, Tegoni M, Freisinger E, Different behavior of the histidine residue toward cadmium and zinc in a cadmium-specific metallothionein from an aquatic fungus, Inorg. Chem 59 (23) (2020) 16988–16997, 10.1021/acs.inorgchem.0c02171. 33205965

[58] Cassandri M, Smirnov A, Novelli F, , Zinc-finger proteins in health and disease, Cell Death Discov. 3 (2017) 17071, 10.1038/cddiscovery.2017.71. 29152378 PMC5683310

[59] Burger N, Mittenbühler MJ, Xiao H, , The human zinc-binding cysteine proteome, Cell 188 (3) (2025) 832–850.e27, 10.1016/j.cell.2024.11.025. 39742810 PMC12120685

[60] Moses D, Guadalupe K, Yu F, , Structural biases in disordered proteins are prevalent in the cell, Nat. Struct. Mol. Biol 31 (2) (2024) 283–292, 10.1038/s41594-023-01148-8. 38177684 PMC10873198

[61] Tesei G, Trolle AI, Jonsson N, , Conformational ensembles of the human intrinsically disordered proteome, Nature 626 (8000) (2024) 897–904, 10.1038/s41586-023-07004-5. 38297118

[62] Boike L, Cioffi AG, Majewski FC, , Discovery of a functional covalent ligand targeting an intrinsically disordered cysteine within MYC, Cell Chem. Biol 28 (1) (2021) 4–13.e17, 10.1016/j.chembiol.2020.09.001. 32966806 PMC7854864

[63] Lee S, Wales TE, Escudero S, , Allosteric inhibition of antiapoptotic MCL-1, Nat. Struct. Mol. Biol 23 (6) (2016) 600–607, 10.1038/nsmb.3223. 27159560 PMC4900187

[64] Yadi Y, Ziyi S, Bin H, , Application of proteomics in the study of toxicology and toxic markers of traditional Chinese medicines, Biomed. Chromatogr 39 (10) (2025) e70216, 10.1002/bmc.70216. 40935568

[65] Liu C, Huang X, Kong J, , Podophyllotoxin mediates hepatic toxicity via the C5a/C5aR/ROS/NLRP3 and cGMP/PKG/mTOR axis in rats based on toxicological evidence chain (TEC) concept by phosphoproteomic analysis, Ecotoxicol. Environ. Saf 289 (2025) 117441, 10.1016/j.ecoenv.2024.117441. 39644570

[66] Cyran AM, Zhitkovich A, HIF1, HSF1, and NRF2: oxidant-responsive trio raising cellular defenses and engaging immune system, Chem. Res. Toxicol 35 (10) (2022) 1690–1700, 10.1021/acs.chemrestox.2c00131. 35948068 PMC9580020

